# Cellulose and *JbKOBITO 1* mediate the resistance of NaHCO_3_-tolerant chlorella to saline-alkali stress

**DOI:** 10.3389/fmicb.2023.1285796

**Published:** 2023-11-15

**Authors:** Jiale Qiu, Jie Zhang, Huihui Zhao, Cuiping Wu, Caoliang Jin, Xiangdong Hu, Jian Li, Xiuling Cao, Shenkui Liu, Xuejiao Jin

**Affiliations:** State Key Laboratory of Subtropical Silviculture, School of Forestry and Biotechnology, Zhejiang A&F University, Hangzhou, China

**Keywords:** algae, cell wall, *Chlamydomonas reinhardtii*, JB17, saccharide metabolism

## Abstract

Carbonate stress has profound impacts on both agricultural and industrial production. Although a number of salinity-tolerant genes have been reported and applied in plants, there is a lack of research on the role of cell wall-related genes in resistance to carbonate. Likewise, in industry, current strategies have not been able to more effectively address the conflict between stress-induced microalgal biofuel accumulation and microalgal growth inhibition. It is of great significance to study the adaptation mechanism of carbonate-tolerant organisms and to explore related genes for future genetic modification. In this study, the role of the cell wall in the NaHCO_3_-tolerant chlorella JB17 was investigated. We found that JB17 possesses a relatively thick cell wall with a thickness of 300–600 nm, which is much higher than that of the control chlorella with a thickness of about 100 nm. Determination of the cell wall polysaccharide fractions showed that the cellulose content in the JB17 cell wall increased by 10.48% after NaHCO_3_ treatment, and the decrease in cellulose levels by cellulase digestion inhibited its resistance to NaHCO_3_. Moreover, the saccharide metabolome revealed that glucose, rhamnose, and trehalose levels were higher in JB17, especially rhamnose and trehalose, which were almost 40 times higher than in control chlorella. Gene expression detection identified an up-regulated expressed gene after NaHCO_3_ treatment, *JbKOBITO1*, overexpression of which could improve the NaHCO_3_ tolerance of *Chlamydomonas reinhardtii*. As it encodes a glycosyltransferase-like protein that is involved in cellulose synthesis, the strong tolerance of JB17 to NaHCO_3_ may be partly due to the up-regulated expression of *JbKOBITO 1* and *JbKOBITO 1*-mediated cellulose accumulation. The above results revealed a critical role of cellulose in the NaHCO_3_ resistance of JB17, and the identified NaHCO_3_-tolerance gene will provide genetic resources for crop breeding in saline-alkali soils and for genetic modification of microalgae for biofuel production.

## 1. Introduction

Carbonate is an important limiting factor for biological growth and for the development of industry and agriculture. On the one hand, carbonate stress not only causes ionic toxicity and osmotic stress, but also further increase the pH of saline-alkaline soils, which seriously affects crop growth and yield (Wang Y. et al., 2020). On the other hand, microalgae, as a potential feedstock for biofuel production (Khoo et al., 2020), has been limited in the commercialisation of microalgae biofuels due to the contradiction between microalgal growth under environmental stress (including carbonate stress) and biofuel accumulation (Ji et al., 2018). Therefore, it is of great significance to study the tolerance mechanism of organisms to carbonate stress and excavate carbonate-tolerant genes to improve the tolerance in plants and industrial microbial strains.

Current research on saline–alkali soils has screened a number of carbonate-tolerant plant resources, with the more familiar ones including *Suaeda corniculata*, *Puccinellia tenuiflora*, oats, etc. (Pang et al., 2016; Bai et al., 2018; Qin et al., 2018; Ye et al., 2019). A range of genes for carbonate tolerance have also been reported. For instance, *SlWRKY28*, a gene cloned from the WRKY family of *Salix linearistipular*, improves the saline-alkali tolerance of transgenic lines by inducing regulatory responses of enzyme genes in the reactive oxygen scavenging pathway (Wang X. et al., 2020). Zheng et al. (2021) reported that *CrPIP23*, a membrane Aquaporins (AQPs) protein gene from *Canavalia rosea*, enhanced alkaline tolerance of transgenic plants by promoting water transport. Liu et al. (2020) reported that a chitinase gene *LcCHI2* from *Leymus chinensis* helped reduce osmotic stress other than Na^+^ ion toxicity or improved water-use-efficiency, improves salt-alkali tolerance in transgenic maize. However, the cell wall, as the first barrier against stress, and its regulatory gene, which has been extensively studied in plant resistance to salt and cadmium stress in recent years (Jia et al., 2021; Dabravolski and Isayenkov, 2023), has not been strongly implicated in resistance to carbonate stress.

Carbonate stress also limits the growth of certain engineered strains for biofuel production. In the industrial field, microalgae are regarded as an important resource for biofuel production. Previous study reported that many environmental stress conditions can promoted cell wall thinning and significantly increased lipid production in *Chlorella vulgaris* (Nadzir et al., 2023). For example, the study by Li et al. (2018) reported that treatment with high concentrations of NaHCO_3_ (160 mM) stimulated the accumulation of *Chlorella vulgaris* lipids, but inhibited cell growth. Therefore, the conflict, between the use of stress to promote biofuel accumulation and stress-induced microbial growth inhibition, limits the industrial production of biofuels in large quantities. Although certain study has reported that product content can be effectively increased by two-stage cultivation of algae, it is simpler and more effective to search for carbonate-tolerant genes and apply them to improve stress resistance (Yun et al., 2019). A recent review summarised the use of genetic modification strategies to improve lipid production in microalgae but these studies have focused on genes that promote lipid synthesis in microalgae (Khoo et al., 2023). Since many genes in algae can be involved in stress response (Hong et al., 2023), the mining of carbonate-tolerant genes is important to optimise the balance between microalgal growth and lipid accumulation.

JB17, is a member of the *Nannochloris* species that we previously isolated from extremely saline-alkali soil in northeastern China. It showed higher tolerance to NaCl and NaHCO_3_, surviving in NaHCO_3_ medium at concentrations up to 1 M (Qiao et al., 2015). JB17, as a eukaryotic microalga, is a unicellular microorganism with both plant and microbial properties. Specifically, it has a structure similar to plant cells, such as a nucleus, chloroplasts and mitochondria, as well as other organelles surrounded by cell membranes, which enable it to photosynthesise to produce high-value bioactive substances (Khoo et al., 2023). In addition, its characteristic of rapid microbial growth enables the response to carbonate stress to be observed in a short period of time, and the unicellular structure makes it easier to study its physiological and molecular responses at the cellular level. Therefore, studying the mechanism of JB17 NaHCO_3_ tolerance and identifying carbonate-tolerant genes will not only benefit agricultural development but also the commercialisation of microalgal biofuels.

In this study, we analyzed the changes in the composition of the cell wall of the saline-alkali tolerant JB17 before and after NaHCO_3_ exposure, and identified a key cell wall gene involved in resistance to NaHCO_3_ through transcriptome analysis and transgenic experiments. Our results not only revealed the mechanism of NaHCO_3_ tolerance in JB17, but also the identified cell wall-related gene would provide valuable genetic resources for agricultural and industrial production.

## 2. Materials and methods

### 2.1. Microalgae strains and culture conditions

The control chlorella (*Chlorella* *variabilis*) and *Chlamydomonas reinhardtii* were obtained from the Freshwater Algae Culture Collection (Institute of Hydrobiology, Chinese Academy of Science, Wuhan, China). The saline-alkali-tolerant *Chlorella* JB17 was previously isolated from extremely alkaline-saline soil (pH > 10) from the Songnen Plain (46°27′N, 125°22′E, Heilongjiang Province, China) (Qiao et al., 2015). For algal growth, JB17 and *C. variabilis* were cultured using Blue-Green Medium (BG11) medium, while *C. reinhardtii* was cultured using Tris-Acetate-Phosphate (TAP) medium. The medium was prepared according to the information provided by the Freshwater Algae Culture Collection at the Institute of Hydrobiology (http://algae.ihb.ac.cn/, accessed on 5 March 2023). All algae were cultured at 23 ± 1°C, with illumination at 40 μM photons m^−1^ s ^−1^ under 16 h light/8 h dark photoperiod (Wang M. et al., 2020).

### 2.2. Transmission electron microscopy

Transmission electron microscopy (TEM) was performed according to previously described methods (Jin et al., 2018). 15 mL algae culture in logarithmic growth phase (at approximately 0.5 × 10^8^ cells/mL) were collected and centrifugated at 800 g for 10 min, and fixed overnight at 4°C in 1 mL fixation buffer (2.5% glutaraldehyde, 0.05 M phosphate, pH 7.2). The glutaraldehyde-fixed algae were washed three times with fixation buffer and then fixed in 2% osmium tetroxide (OsO_4_) at 4°C for 2 h in the dark. Then the algae were dehydrated through a series of graded ethanol (70, 80, 90, 95, and 100%) for 10 min each time, and then embedded in Spurr’s resin. Ultrathin sections (70 nm) were cut with an ultramicrotome (EM UC7; Leica, Germany) and sequentially stained with uranyl acetate for 20 min and Reynolds’ lead citrate for 5 min. The specimens were observed using a Hitachi H-7650 (Hitachi, Japan) or a JEM-1230 (JEOL Co. Ltd., Japan) transmission electron microscope operated at 80 kV.

### 2.3. Plasmid construction

Firstly, total RNA was extracted from JB17, and mRNA was reverse transcribed into cDNA using M-MLV reverse transcription kit (Thermo Fisher, Shanghai, China). The full-length coding sequence (CDS) of *JbKOBITO 1* was amplified from cDNA and inserted into the *Hpa* I-linearized plasmid pLM006 by homologous recombination methods to generate a vector for electro-transformation of *C. reinhardtii*. The pLM006 plasmid was expressed under the control of the PASD promoter, and mCherry protein was expressed at the C-terminus of the target gene. The primers used for plasmid construction are listed in Supplementary Table S1. The correctness of the plasmid was verified by DNA sequencing.

### 2.4. Expression analysis of *JbKOBITO 1*

The expression changes of *JbKOBITO 1* before and after NaHCO_3_ treatment were detected by RT–qPCR as described previously (Li et al., 2023). The primers used are listed in Supplementary Table S1.

### 2.5. Nuclear transformation of *Chlamydomonas reinhardtii* by electroporation

The generation of transgenic algae lines overexpressing *JbKOBITO 1* was performed as described previously (Li et al., 2023). After the colonies had grown, different primers were designed to identify the mcherry fusion fluorescent protein gene, the PASD promoter and the target gene by PCR, to obtain the positive strains into which the plasmid had been successfully transferred. Three overexpression lines were analyzed for their NaHCO_3_ tolerance, and the results showed the same trends.

### 2.6. Cell wall component extraction and detection

Log-phase cells were aspirated and added to a modified TAP liquid medium containing 0 mM and 300 mM NaHCO_3_ for 3 days. Then, the cells were cultured in the dark for 12 h to consume starch before being rapidly frozen in liquid nitrogen, after which the cells were disrupted to obtain the cell wall by milling using a steel bead and FastPrep-24^™^ 5G bead-beating grinder (MP Biomedicals, United States). All the cells were examined under a microscope for disruption to ensure that the cell wall preparation was as clean as possible. The cell samples were added to 80% (vol/vol) ethanol, extracted on ice and the supernatant was removed by centrifugation. This was repeated until a clean sample was obtained. Samples were then extracted with acetone for 10 min at room temperature, and the supernatant was removed by centrifugation. Methanol was extracted for 10 min, and the supernatant was removed by centrifugation to obtain the final white substance as a cell wall. The samples were dried in an oven, the weights were recorded, and the dried cell walls were sent to Wuhan ProNets Biotechnology Co., Ltd. (Wuhan, China, http://www.pronetsbio.com, accessed on 20 March 2023) for detection. The experiments were repeated three times.

### 2.7. Cell wall saccharide metabolome detection

*Chlorella variabilis* and JB17 were cultivated in BG11 for 7 days, and then diluted to the fresh BG11 medium with 0 mM or 300 mM NaHCO_3_, respectively. After 3 days of treatment, the cells were treated in the dark for 12 h to consume starch before collection, and then sent to Wuhan Metwell Biotechnology Co., Ltd. (Wuhan, China, http://www.metware.cn, accessed on 25 March 2023) for monosaccharide or disaccharide detection.

### 2.8. Cellulase digestion assays

JB17 was cultured in BG11 liquid medium for 7 days and then were collected by centrifugation. Each gram of algae was treated with 0, 50 and 100 U cellulase (Solarbio, Beijing, China) for 3 days. The cultures with a cell density of approximately 1 × 10^8^ cells/mL, were then gradient diluted in 96-well plates with sterile water and 4 μL of the dilutions were spotted in modified BG11 solid medium supplemented with 0, 50 and 100 mM NaHCO_3_, respectively. After 16 days of culture, algal growth was observed.

### 2.9. Determination of NaHCO_3_ tolerance of transgenic *Chlamydomonas reinhardtii*

Log-phase *C. reinhardtii* cells (at approximately 0.5 × 10^8^ cells/mL) were collected, serially diluted and spotted onto the modified TAP solid medium containing 0 or 40 mM NaHCO_3_, respectively. After growth for about 10 days, algal growth was observed. For the growth curve determination, the log-phase *C. reinhardtii* cells were diluted to an OD_680_ of 0.15 in TAP medium containing 0 mM, 35 mM, and 40 mM NaHCO_3_, respectively, and the cell density of the cultures was determined using a spectrophotometer (Ultrospec 2,100 Pro, Biochrom, St. Albans, UK), at different time points from 0 to 120 h.

### 2.10. Accession numbers

Sequence data of glycosyltransferase-like KOBITO 1 from different organisms in this work can be found in NCBI under the following accession number: *Nannochloris* sp. JB17 (OR486971), *Arabidopsis thaliana* (NP187467.1), *Chlorella vulgaris* (KAI3434462.1), *Micractinium conductrix* (PSC67501.1), *Chlorella* *variabilis* (XP005843966.1), *Chlorella sorokiniana* (PRW20664.1), *Coccomyxa* sp. Obi (BDA46538.1), *Oryza sativa* Japonica Group (NP001393036), and *Capsicum annuum* (PHT71472.1).

## 3. Result

### 3.1. *Nannochloris* sp. JB17 has a more stable ultrastructure under NaHCO_3_ stress compared to the control

**Figure 1:**
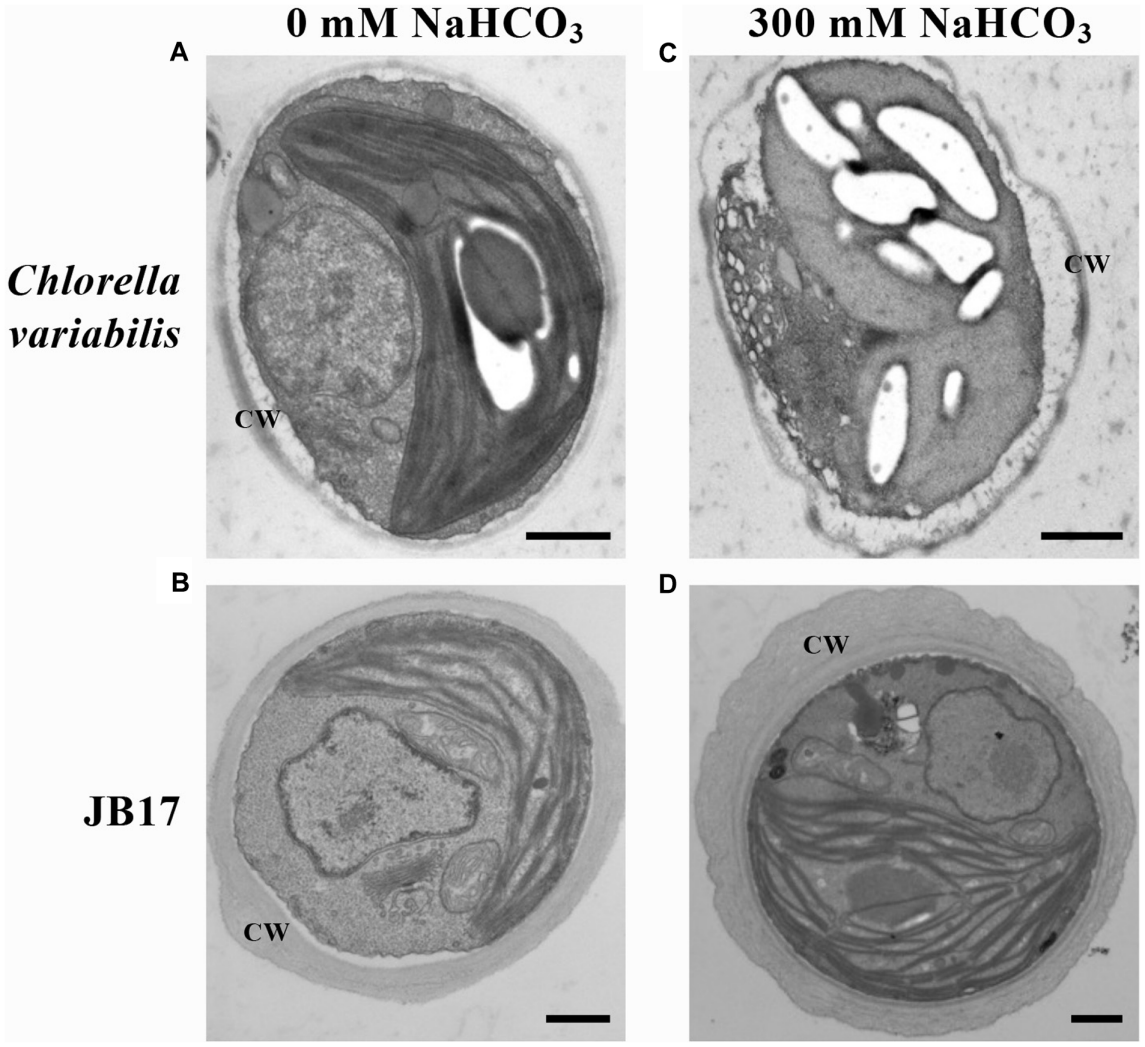
Electron microscopic visualization of *C. variabilis* and JB17 exposed to NaHCO_3_ stress. **(A,B)** Untreated *C. variabilis* and JB17 were used as controls. **(C,D)** Representative images of the ultrastructure of *C. variabilis* and JB17 treated with NaHCO_3_. CW, cell wall; scale bars, 500 nm.

In order to study the ultrastructural changes of JB17 before and after treatment with high concentrations of NaHCO_3_, the morphology was observed using TEM. It showed that under normal conditions, the control chlorella and JB17 had intact cellular structures (Figures 1A,B). Interestingly, the cell wall of JB17 was thicker, with a thickness of about 300–600 nm, while that of the control chlorella was only about 100 nm thick (Figures 1A,B). After 300 mM NaHCO_3_ treatment, the cell wall morphology of JB17 was similar to that before stress, whereas the cell wall of the control chlorella was deformed and damaged, and the internal structure of the cell was affected to some extent (Figures 1C,D). These results suggest that the stable and thick cell walls of JB17 may be involved in its tolerance to high concentrations of NaHCO_3_.

### 3.2. Changes in JB17 cell wall composition before and after NaHCO_3_ treatment

The composition of cell wall polymers usually determines the cell wall structure. Plant cell walls are mainly composed of cellulose, hemicellulose, pectin, and lignin (Lampugnani et al., 2018). Many studies have reported that changes in these components are involved in plant resistance to salt stress (Dabravolski and Isayenkov, 2023), but the role of these components in resistance to carbonate stress is unclear. Therefore, in order to elucidate the changes in JB17 cell wall components in response to NaHCO_3_ stress, the contents of major polysaccharide components including cellulose, hemicellulose, lignin, and pectin of JB17 cell walls before and after NaHCO_3_ treatment were measured. When exposed to 300 mM NaHCO_3_, the levels of all four components changed, with the exception of lignin, which did not change significantly (Table 1). Of these, cellulose content was the highest as a percentage of dry weight and showed the greatest percentage change in response to NaHCO_3_ treatment. The cellulose content was significantly increased by 10.48% compared to that of 0 mM NaHCO_3_ (Table 1). These results suggest that JB17 may adapt to NaHCO_3_ stress by altering the polysaccharide composition of the cell wall.

**Table 1:** Changes in polysaccharides in JB17 cell walls exposed to NaHCO_3_ stress.

Constituent	Content (mg/g)				Ralative content
	0 mM NaHCO_3_	Mean ± SD	300 mM NaHCO_3_	Mean ± SD	
Cellulose	340.82	341.35 ± 4.37	371.32	377.14 ± 5.07	110.48%^**^
	337.27		379.55		
	345.96		380.56		
Hemicellulose	254.45	250.71 ± 5.09	229.79	233.49 ± 4.72	93.13%^*^
	244.91		231.87		
	252.78		238.80		
Lignin	78.33	76.68 ± 2.69	79.78	79.34 ± 0.95	103.47%^ns.^
	73.57		78.25		
	78.13		79.98		
Protopectin	2.02	2.02 ± 0.03	1.84	1.85 ± 0.01	91.58%^**^
	1.99		1.86		
	2.04		1.86		
Soluble pectin	2.02	2.00 ± 0.02	1.79	1.81 ± 0.02	90.50%^**^
	1.99		1.83		
	1.98		1.82		

ns., no significant difference; **, *p* < 0.01, *, 0.01 < *p* < 0.05, Student’s *t*-test.

### 3.3. Cellulose content affects JB17 tolerance to NaHCO_3_

**Figure 2:**
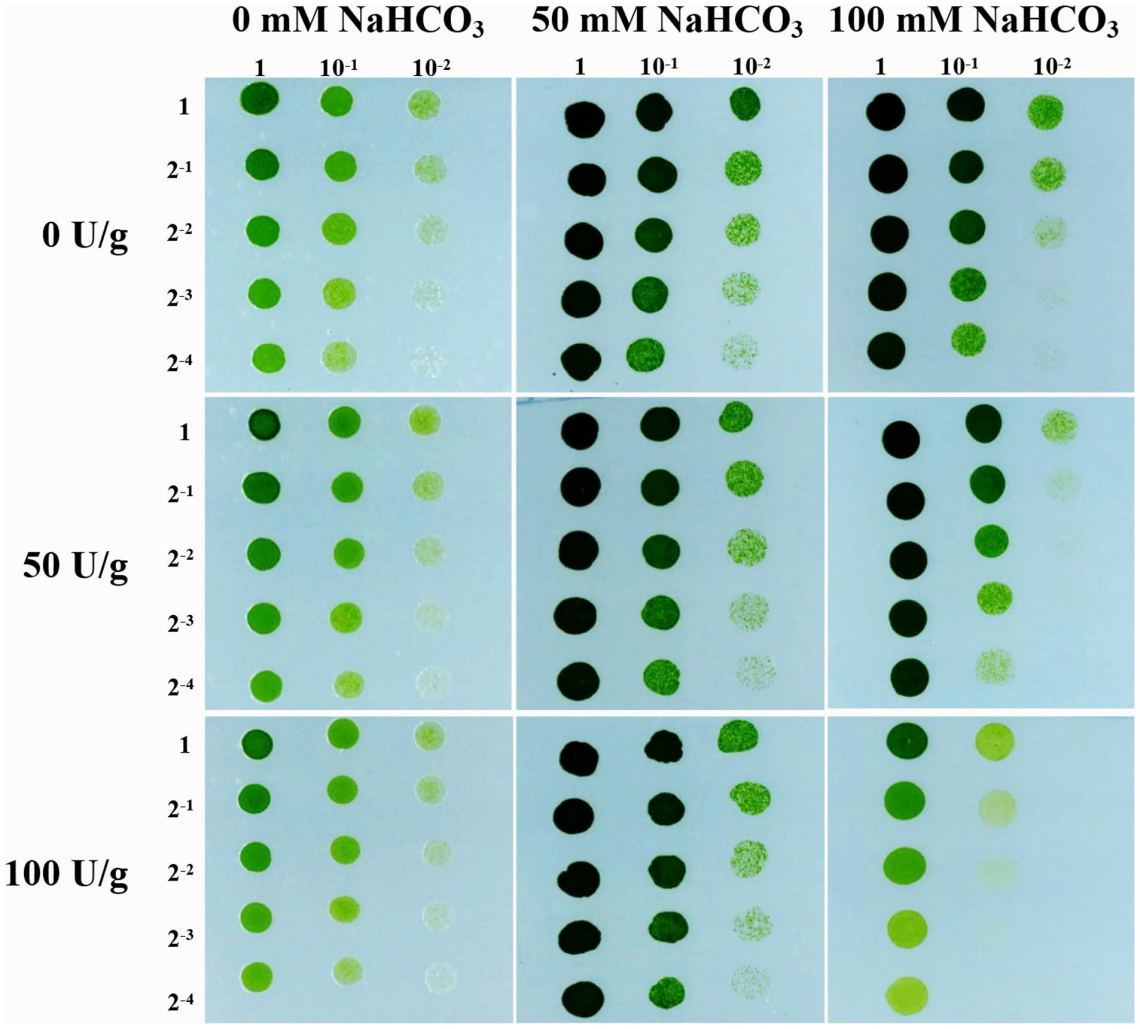
Cellulose content affects JB17 tolerance to NaHCO_3_. The treatment without cellulase addition was used as control. Each treatment was carried out in three biological replicates. The numbers labelled in the figure are the dilution multiples for the gradient dilution assays.

Previous study has found that the cell wall cellulose content of rice increased by 10.64% under drought stress, thereby enhancing the drought tolerance of rice (Sun et al., 2022). Therefore, we followed up with a cellulase digestion assay to test whether the accumulated cellulose in JB17 cell wall is critical for NaHCO_3_ tolerance. Under the treatment of different concentrations of cellulase, there were no significant differences in the growth of JB17 on 0 and 50 mM NaHCO_3_ plates, indicating that cellulase digestion did not affect the normal growth of algae (Figure 2). When JB17 was grown on 100 mM NaHCO_3_ plates, the growth of 50 U/g cellulase-treated algae showed a slight degree of inhibition, and the growth defects were more pronounced when the concentration of cellulase reached 100 U/g (Figure 2). In addition, the results of cellulose content determination showed that the cellulose content of JB17 cell wall decreased significantly by 18.472% after 100 U/g cellulase treatment (Supplementary Figure S1). These results indicate that the decrease in cellulose content of JB17 affects its tolerance to high concentrations of NaHCO_3_ stress, which confirms our hypothesis that NaHCO_3_-induced cellulose accumulation in the cell wall contributes to the tolerance of JB17 to NaHCO_3_.

### 3.4. Effect of NaHCO_3_ on saccharide metabolism in JB17

**Figure 3:**
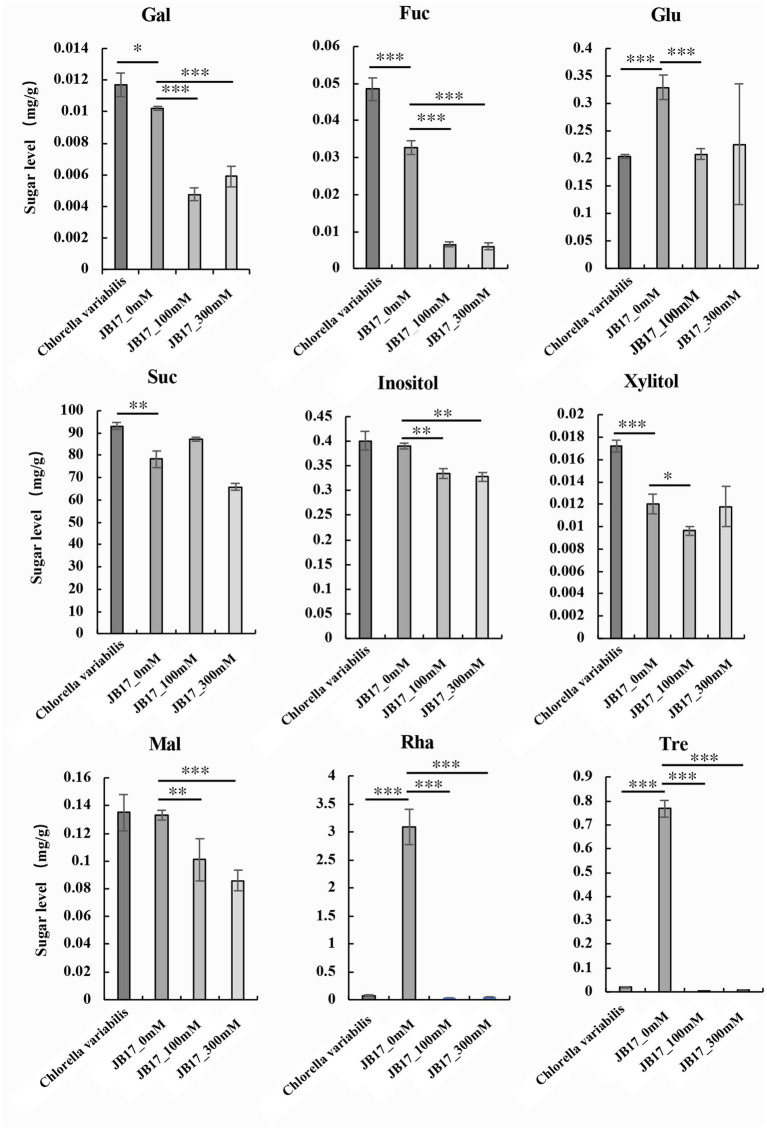
Changes in saccharide metabolism in JB17 before and after NaHCO_3_ treatment. Gal, galactose; Fuc, fucose; Glu, glucose; Suc, sucrose; Mal, maltose; Rha, rhamnose; Tre, trehalose. Error indicates standard deviation (SD) from three independent experiments. ***p < 0.001, **p < 0.01, *0.01 < *p* < 0.05, Student’s *t*-test.

Metabolites are downstream products of the genome and final products (Qiu et al., 2023). They reflect the final changes in biological systems when they are subjected to genetic and environmental alterations (Alawiye and Babalola, 2021). Changes in cell wall polysaccharides are closely related to saccharide metabolism. Therefore, we further studied the changes in saccharide metabolism in JB17 before and after NaHCO_3_ treatment. We determined nine monosaccharide and disaccharide components in *C.* *variabilis* without NaHCO_3_ treatment, and JB17 treated with 0 mM, 100 mM, and 300 mM NaHCO_3_, including galactose, fucose, sucrose, glucose, inositol, xylitol, maltose, rhamnose, and trehalose. Under untreated NaHCO_3_ conditions, sucrose was the major component of both JB17 and the control chlorella, and galactose, fucose, xylitol, and sucrose were lower in JB17 cell walls than the control chlorella. In contrast, glucose, rhamnose, and trehalose in JB17 were higher, especially rhamnose and trehalose, which were nearly 40-fold higher than the control chlorella (Figure 3). After NaHCO_3_ treatment, the sucrose in JB17 did not change significantly, while other eight saccharide components, including galactose, fucose, glucose, inositol, xylitol, maltose, rhamnose, and trehalose, showed significant decreases, among which rhamnose and trehalose showed the most significant changes with a decrease of about 100-fold (Figure 3). These saccharide fractions were able to undergo significant changes in response to NaHCO_3_, and their metabolic processes may be involved in the cell wall polysaccharide changes and NaHCO_3_ adaptation in JB17.

### 3.5. NaHCO_3_ stress induces up-regulation of *JbKOBITO 1* expression

To search for key genes that respond to NaHCO_3_ stress and improve NaHCO_3_ tolerance, we analyzed the transcriptome sequencing data of JB17 cells after 0 mM and 300 mM NaHCO_3_ treatment (data not shown). Among the genes whose expressions were up-regulated by 300 mM NaHCO_3_, a gene involved in saccharide metabolism and related to cell wall synthesis was identified. And a search for its homologous proteins with known functions in the Uniprot protein database (https://www.uniprot.org/, accessed on 15 July 2023) revealed that it shares 55% amino acid sequence identity with the *A. thaliana* glycosyltransferase-like protein KOBITO 1 (Gen Bank NP 187467.1). Therefore, it was tentatively named *JbKOBITO 1*.

**Figure 4:**
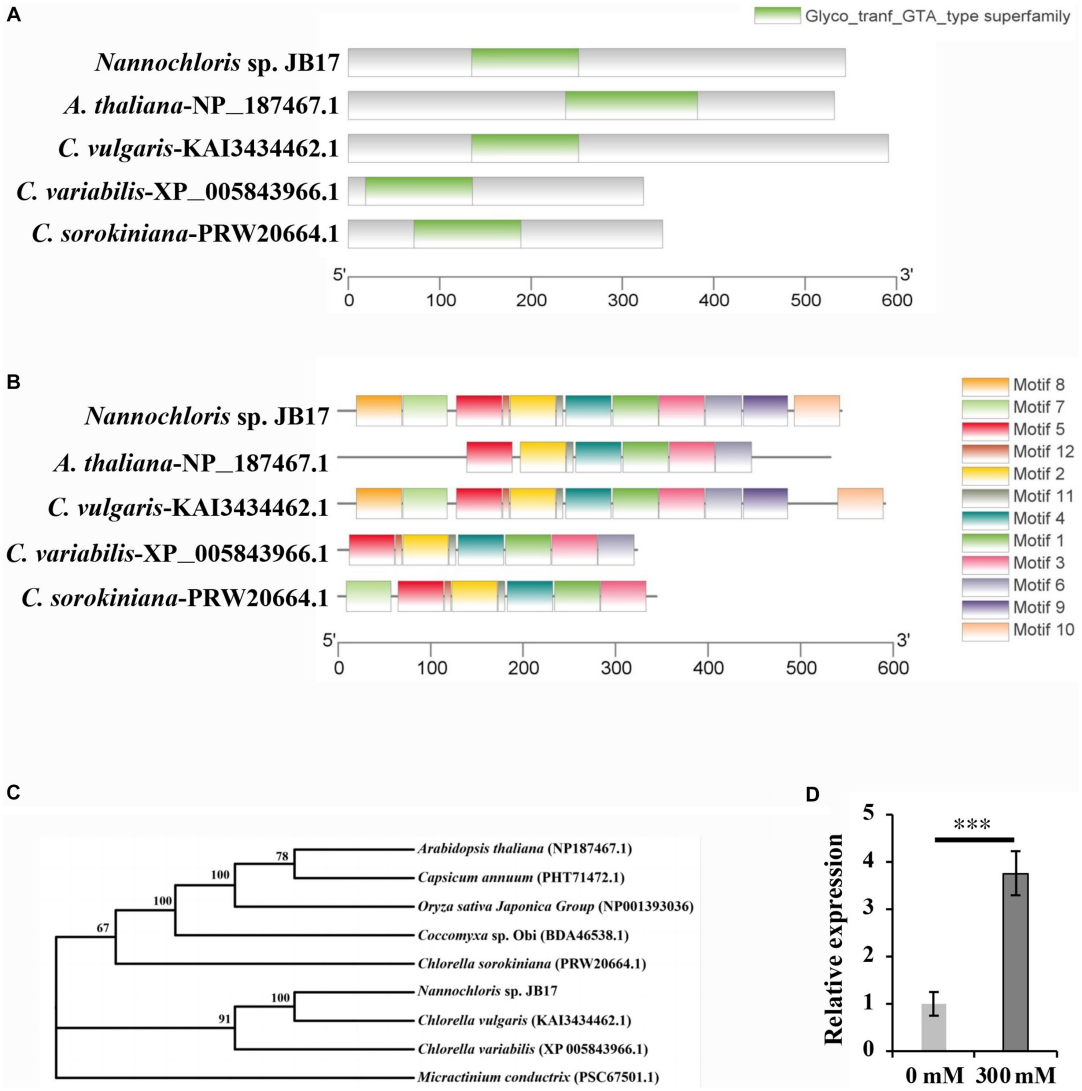
Sequence analysis and expression detection of JbKOBITO 1. **(A)** JbKOBITO 1 possesses a conserved Glyco_tranf_GTA_type superfamily domain. **(B)** Conserved motif analysis of glycosyltransferase-like protein KOBITO 1 from algae and *A. thaliana*. The different colored boxes represent different motifs and their position in each protein sequence. All motifs were identified by MEME. **(C)** Neighbor-joining (NJ) phylogenetic relationships of the KOBITO 1 protein from JB17 and predicted homologs from other species or organisms. Bootstrap values were calculated 1,000 times; values <50% are not shown. **(D)** The expression of *JbKOBITO 1* was induced by NaHCO_3_ treatment. Error bars indicate standard deviation (SD). ***, *p* < 0.001.

The amino acid sequence of the glycosyltransferase-like KOBITO 1 from *Nannochloris* sp. JB17, *C. vulgaris*, *C.* *variabilis*, *C. sorokiniana*, and *A. thaliana* were analyzed using NCBI Batch CD-search (https://www.ncbi.nlm.nih.gov/Structure/bwrpsb/bwrpsb.cgi, accessed on 15 July 2023). MEME software (https://meme-suite.org/meme/tools/, accessed on 15 July 2023) was used to identify conserved motifs of glycosyltransferase-like KOBITO 1 from different organisms. All these proteins shared a conserved Glyco_tranf_GTA_type superfamily domain (Figure 4A), which is involved in synthesizing and modifying of saccharide molecules. Comparing with JbKOBITO 1 in JB17, the sequences of the homologous proteins in *C. variabilis* and *C. sorokiniana* were much shorter in size, and the homologous protein sequences in *A. thaliana* and *C. vulgaris* were similar in size, but lacked the motif 7, 8, 9, 10, 12 in *A. thaliana* (Figure 4B).

Evolutionary tree was constructed using MEGA 11 software. It was clear that the evolutionary relationship between JbKOBITO 1 and *C. vulgaris* hypothetical protein (D9Q98_002539) was close (Figure 4C). Sequence alignment was performed using the software Jalview (http://www.jalview.org/, accessed on 16 July 2023), and Muscle with Defaults was selected to align. Some highly conserved sites were found (Supplementary Figure S2). Therefore, JbKOBIOT 1 may have a similar function to the *Arabidopsis* glycosyltransferase-like protein KOBITO 1, which is involved in the coordination between cell elongation and cellulose synthesis by promoting the expression of related genes (Pagant et al., 2002). Moreover, to elucidate whether NaHCO_3_ stress up-regulated the expression of *JbKOBITO 1*, the transcript levels of *JbKOBITO 1* were tested by RT-qPCR. And the results showed that *JbKOBITO 1* was significantly up-regulated in the context of 300 mM NaHCO_3_ treatment, which was consistent with the results of the transcriptome (Figure 4D). It prompted us to speculate that the NaHCO_3_-induced up-regulation of *JbKOBITO 1* may be involved in the synthesis of accumulated cellulose in the JB17 cell wall.

### 3.6. Overexpression of *JbKOBITO 1* improves NaHCO_3_ tolerance of transgenic *Chlamydomonas reinhardtii*

**Figure 5:**
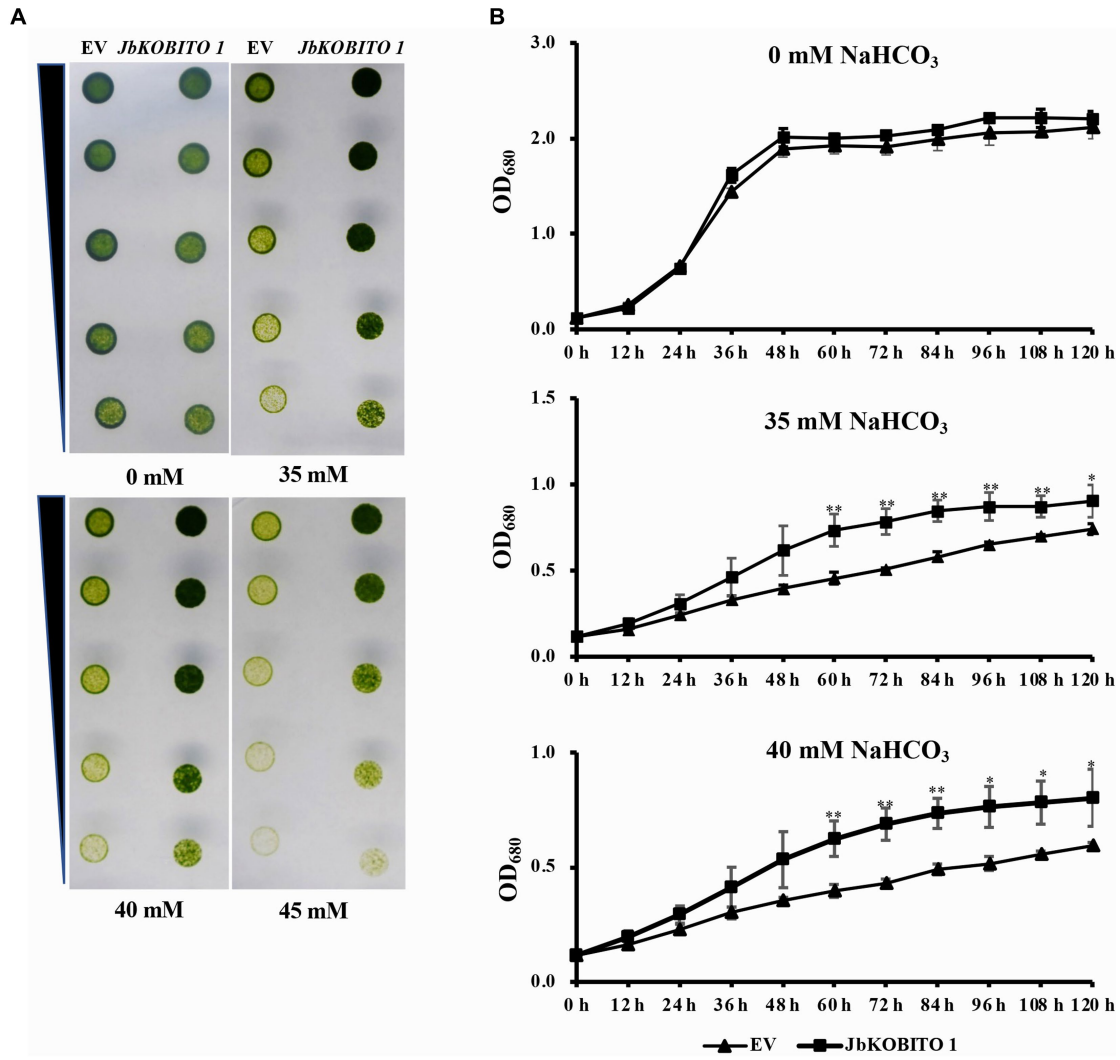
Overexpression of *JbKOBITO 1* enhanced *C. reinhardtii* NaHCO_3_ tolerance. **(A)** Gradient dilution assays of transgenic *C. reinhardtii* on solid medium with different concentrations of NaHCO_3_. **(B)** Growth curve of transgenic *C. reinhardtii* in liquid medium with different concentrations of NaHCO_3_. **p < 0.01, *0.01 < *p* < 0.05, Student’s *t*-test.

In order to verify whether *JbKOBITO 1* is involved in NaHCO_3_ tolerance, the recombinant plasmid expressing *JbKOBITO 1* was electroporated into *C. reinhardtii*. Then, the NaHCO_3_ tolerance of the positively transformed strains was monitored. Gradient dilution assays showed no significant difference in the growth of *C. reinhardtii* overexpressing the empty vector and overexpressing the target gene on 0 mM NaHCO_3_ plate, indicating that *JbKOBITO 1* overexpression did not affect the growth of algae (Figure 5A). With the increase of NaHCO_3_ concentration, the growth of the control was significantly inhibited, whereas the growth of *C. reinhardtii* overexpressing *JbKOBITO 1* was significantly better than that of the control (Figure 5A). Similarly, the sensitivity of transgenic *C. reinhardtii* to different concentrations of NaHCO_3_ in liquid medium was consistent with the results from gradient dilution assays. The cell density of *C. reinhardtii* expressing the empty vector was not significantly different from that of *C. reinhardtii* expressing *JbKOBITO 1* in the absence of NaHCO_3_ (Figure 5B). However, at NaHCO_3_ concentrations of 35 mM, the cell density of *C. reinhardtii* expressing *JbKOBITO 1* was significantly higher than that of the control group from 60 h, with final cell densities (120 h) of 0.903 and 0.740, respectively (Figure 5B). When the concentration of NaHCO_3_ was 40 mM, the difference was more obvious, with final cell densities (120 h) of 0.802 and 0.594, respectively (Figure 5B). These results demonstrated that the introduction of *JbKOBITO 1* into algae could improve the ability of *C. reinhardtii* to resist NaHCO_3_.

## 4. Discussion

Plant cell walls play an important role in sensing and responding to stress. Recent studies have suggested that the plant cell wall is highly dynamic, with changes in structure and composition occurring during plant growth and development or in response to abiotic stresses to maintain normal life activities (Le Gall et al., 2015; Jia et al., 2021). In order to investigate the role of the JB17 cell wall in carbonate tolerance, we first observed its ultrastructure by TEM, and found that it has a thicker cell wall than the control chlorella, and the morphology and structure of the cell are still stable under the high concentration of carbonate stress. Previous studies have found that salt-adapted cells of *A. thaliana* acquire a thicker cell wall structure than control cells, which physically strengthens the cells, provides better mechanical strength, reduces salt ion invasion and enhances tolerance to salt stress (Chun et al., 2019). Nadzir et al. (2023) also found that the cell wall thickness of *Chlorella vulgaris* increased from 175 nm to 321 nm at higher salt salinity. Therefore, the ability of JB17 to tolerate high concentrations of NaHCO_3_ may be closely related to its cell wall.

Subsequently, we examined the changes in the main components of the cell wall before and after treatment with NaHCO_3_, which showed a significant increase in cellulose content, and a significant decrease in hemicellulose, soluble pectin and protopectin content. But of these, the change in cellulose content is the most pronounced, as cellulose content was the highest as a percentage of dry weight and showed the greatest percentage change in response to NaHCO_3_ treatment. This suggests that carbonate stress induces complex cell wall polysaccharide modifications and that the ability of JB17 to tolerate carbonate stress may be closely related to changes in cellulose. It is consistent with recent research advances which have demonstrated the impact of cellulose synthesis on plant tolerance to abiotic stresses. For example, DROUGHT1 (DROT1) encodes a COBRA-like protein that confers drought resistance in rice. It has been shown that under drought stress, DROT1 enhances drought tolerance in rice by regulating cell structure through increasing cellulose content by 10.64% and maintaining cellulose crystallinity (Sun et al., 2022). Moreover, Liu et al. reported that the cellulose content in the cell wall of the halophyte *Suaeda salsa* was significantly higher than that of the glycophyte *Spinacia oleracea* in a high-salinity environment, giving the cell wall a relatively high hardness, which resisted the deformation of the cell wall under this pressure, and protected the stability of the cell wall structure under salinity stress (Liu et al., 2022). Our results of a cellulase digestion assay also indicated that the decrease in cellulose content of JB17 affects its tolerance to high concentrations of NaHCO_3_.

Previous studies have also reported on the relationship between the content of pectin and the resistance to stress. An et al. (2014) observed that salt-tolerant soybean varieties contained higher pectin content than salt-sensitive varieties. Jin et al. (2020) reported that *OsEIL2* (ethylene insensitive 3-like2) regulates the *β*-subunit of the polygalacturonase (PG1*β*-like) subfamily of genes in rice *OsBURP16*. Overexpression of *OsEIL2* in rice resulted in lower pectin content and reduced salt stress tolerance. In these studies, higher pectin levels appeared to favor plant salt tolerance. In addition, pectin was also found to undergo structural changes under the action of modifying enzymes, affecting cell wall characteristics and further affecting its ability to cope with salt stress (Yan et al., 2018). However, in our study, protopectin and soluble pectin content decreased under NaHCO_3_ stress_._ This may be due to the different stress response mechanisms of pectin in higher plants and microorganisms. Alternatively, changes in pectin content in JB17 did not play an important role in stress tolerance, as evidenced by the fact that the pectin content in higher plant cell walls is up to 40% (Dabravolski and Isayenkov, 2023), whereas the JB17 cell wall contained very little pectin.

In order to identify the key genes involved in NaHCO_3_ tolerance, transcriptome data of JB17 before and after NaHCO_3_ treatment were analyzed. *JbKOBITO 1*, whose expression was up-regulated in response to NaHCO_3_, caught our attention. It is homologous to the *A. thaliana* glycosyltransferase-like protein KOBITO 1 and has a conserved Glyco_tranf_GTA_type superfamily domain. Previous studies have reported that the functions of this protein include coordinating cell elongation and cellulose synthesis by promoting the expression of genes involved in cell elongation and cellulose synthesis, acting as a regulator of intercellular junction filament permeability, and mediating the saccharide response essential for ABA and growth (Pagant et al., 2002; Dinneny et al., 2008; Kong et al., 2012; Wang et al., 2015). We found that both the expression of *JbKOBITO 1* and the cellulose content in JB17 cell wall were significantly up-regulated after NaHCO_3_ treatment, and the overexpression of *JbKOBITO 1* in *C. reinhardtii* can enhance the tolerance of *C. reinhardtii* to NaHCO_3_. Therefore, we speculated that the strong tolerance of JB17 to NaHCO_3_ may be partially attributed to the *JbKOBITO 1*-mediated cellulose accumulation under NaHCO_3_ stress. Of course, it cannot be excluded that other genes are also involved in this process. For example, Tang et al. (2022) reported a gene *OsUGE3* in rice, which exhibits UDP-galactose/glucose epimerase activity and provides substrate for cellulose synthesis, thereby promoting cellulose accumulation. *OsUGE3*-mediated increase in cellulose accumulation enhances rice tolerance to salt stress by means of cellular bulking that maintains the integrity of the cell wall. Therefore, there may be other genes involved in JB17 cellulose synthesis, although no other cellulose synthesis-related genes with significant up-regulation in expression were found in our transcriptome data, they may function through post-translational modification or changes in enzyme activity.

Changes in polysaccharides are closely related to saccharide metabolism, which prompted us to investigate JB17 saccharide metabolism. Our results showed higher levels of JB17 glucose, rhamnose and trehalose compared to the NaHCO_3_ low-tolerance control chlorella, especially rhamnose and trehalose, which are nearly 40 times higher than the control chlorella. Since the discovery of the gene family encoding active trehalose phosphate synthase (TPS; EC 2.4.1.15) and trehalose phosphatases (TPP; EC 3.1.3.12) in *A. thaliana*, the metabolism of trehalose has been recognized as playing an essential and pervasive role in the life of plants (Lunn et al., 2014). Trehalose (Tre), a non-reducing disaccharide, is an excellent osmolyte for inducing salt tolerance (Ye et al., 2023). Joshi et al. (2020) reported that transgenic lines overexpressing trehalose biosynthetic fusion gene (TPSP) in rice had higher trehalose accumulation and enhanced tolerance to salt and alkali stress. Exogenously applied Tre increases endogenous Tre, another strategy to reduce the adverse effects of salt stress (Kosar et al., 2019). Several studies have reported that exogenous application of trehalose improves salt tolerance in plants (Yuan et al., 2022; Yang et al., 2022a,b). Siddiqui et al. (2020) reported that endogenous glucose content increases when plants are subjected to various abiotic stresses as well as exogenous supply of glucose. Ghorbani Javid et al. (2011) suggested that glucose has a direct role in osmoregulation and radical scavenging in rice grains under salt stress. All these studies suggest that the accumulation of trehalose and glucose may play an important role in plant salt tolerance. This is consistent with our study in which JB17 had a higher trehalose and glucose content. Although there is a lack of research on the role of rhamnose in plant resistance to saline and alkaline stress, it has been found in some studies that rhamnose is beneficial in maintaining osmoregulation and enhancing tolerance to osmotic stress in plants (Ghouili et al., 2021).

Taken together, our study provides an experimental basis for understanding the role of the cell wall polysaccharide, especially the role of cellulose in resistance to carbonate stress. The obtained NaHCO_3_ tolerance gene *JbKOBITO 1* will provide genetic resources for crop breeding in saline-alkali soils and for genetic modification of strains for biofuel production. In the future, genetic modification strategies can be used to increase the cellulose content in the cell walls of crops to increase tolerance to saline-alkali stress for agricultural development, as well as to solve the problem of microalgae that accumulate more lipids under NaHCO_3_ stress but whose growth is inhibited.

## Data availability statement

The datasets presented in this study can be found in online repositories. The names of the repository/repositories and accession number(s) can be found in the article/Supplementary material.

## Author contributions

JQ: Formal analysis, Investigation, Methodology, Writing – original draft. JZ: Formal analysis, Investigation, Methodology, Writing – review & editing. HZ: Formal analysis, Investigation, Methodology, Writing – review & editing. CW: Formal analysis, Investigation, Methodology, Writing – review & editing. CJ: Formal analysis, Investigation, Methodology, Writing – review & editing. XH: Formal analysis, Investigation, Methodology, Writing – review & editing. JL: Writing – review & editing, Data curation, Investigation. XC: Writing – review & editing, Funding acquisition. SL: Conceptualization, Writing – review & editing, Resources. XJ: Conceptualization, Formal analysis, Funding acquisition, Investigation, Methodology, Writing – review & editing.
