# Supplementary material for: Cellulose and JbKOBITO 1 mediate the resistance of NaHCO3-tolerant chlorella to saline-alkali stress
Source: Front Microbiol. 2023 Nov 15;14:1285796. doi: 10.3389/fmicb.2023.1285796 (PMC10684911; doi:10.3389/fmicb.2023.1285796)
Supplement: Supplementary file 1 [file Table_1.docx]

**Supplementary Table 1** Primers used in this study.

| **Name** | **Primer sequence (5'-3')** | **Purpose** |
| --- | --- | --- |
| JbKOBITO 1-1 | ATGAGCGCCCGCGGGCCATTG | For amplification of full-length *JbKOBITO 1* from cDNAs |
| JbKOBITO 1-2 | TCAAGCGTGCCCTGCTGCG |  |
| JbKOBITO 1-3 | GCTACTCACAACAAGCCCAGTTATGAGCGCCCGCGGGCCATTG | For insertion of full-length *JbKOBITO 1* into pLM006 using homologous recombination |
| JbKOBITO 1-4 | GAGCCACCCAGATCTCCGTTAGCGTGCCCTGCTGCG |  |
| JbKOBITO 1-5 | CATGAGGTAGTAGGCACGC | For PCR detection of transgenic *Chlamydomonas reinhardtii* |
| JbKOBITO 1-6 | GGTCTTCCACATCATCCCG |  |
| mCherry-1 | AGGGCGAGGAGGATAACAT |  |
| mCherry-2 | TCTTGACCTCAGCGTCGTA |  |
| PASD-1 | ATGCCTCAACAACCCGTA |  |
| PASD-2 | TTGTTGTGAGTAGCAGTGGG |  |
| JB17- ACTIN-1 | TCCGTCTGGCAGTTCAAAG | For RT-qPCR assay |
| JB17- ACTIN-2 | TGCGTGACATCAAGGAGAAG |  |
| JbKOBITO 1-7 | TTACGGGAACGGAAAGTCAG |  |
| JbKOBITO 1-8 | ATGAGCTCGGTGTCGTTCTT |  |
