# Supplementary material for: Cellulose and JbKOBITO 1 mediate the resistance of NaHCO3-tolerant chlorella to saline-alkali stress
Source: Front Microbiol. 2023 Nov 15;14:1285796. doi: 10.3389/fmicb.2023.1285796 (PMC10684911; doi:10.3389/fmicb.2023.1285796)
Supplement: Supplementary file 2 [file Image_1.pdf]

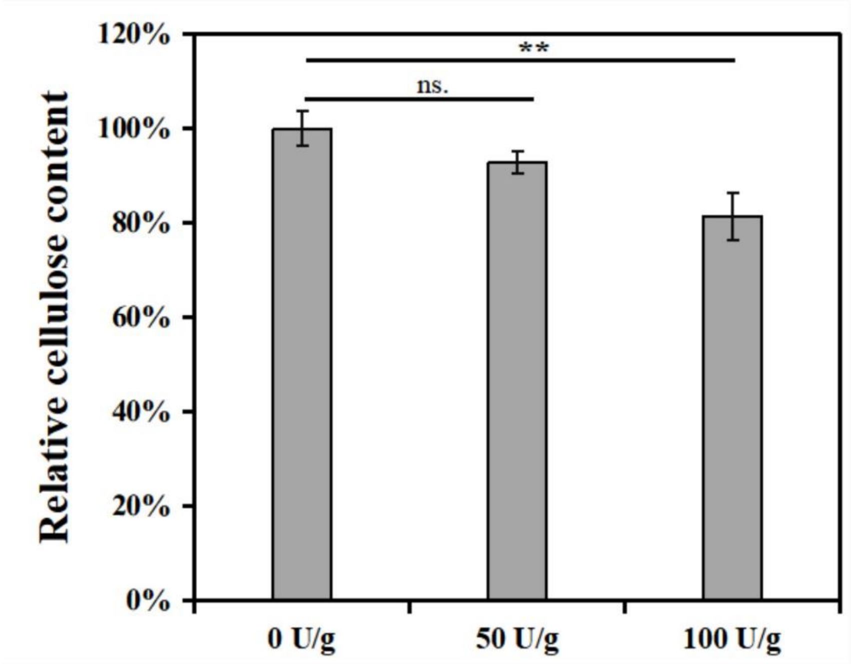

Supplementary Figure 1 Changes in cellulose content of JB17 cell wall under cellulase treatment. The vertical axis represents the relative polysaccharide content. Error bars indicate standard deviation (SD). ns., no significant difference; **, $p < 0.01$, Student's t-test.
