# Supplementary material for: Cellulose and JbKOBITO 1 mediate the resistance of NaHCO3-tolerant chlorella to saline-alkali stress
Source: Front Microbiol. 2023 Nov 15;14:1285796. doi: 10.3389/fmicb.2023.1285796 (PMC10684911; doi:10.3389/fmicb.2023.1285796)
Supplement: Supplementary file 3 [file Image_2.pdf]

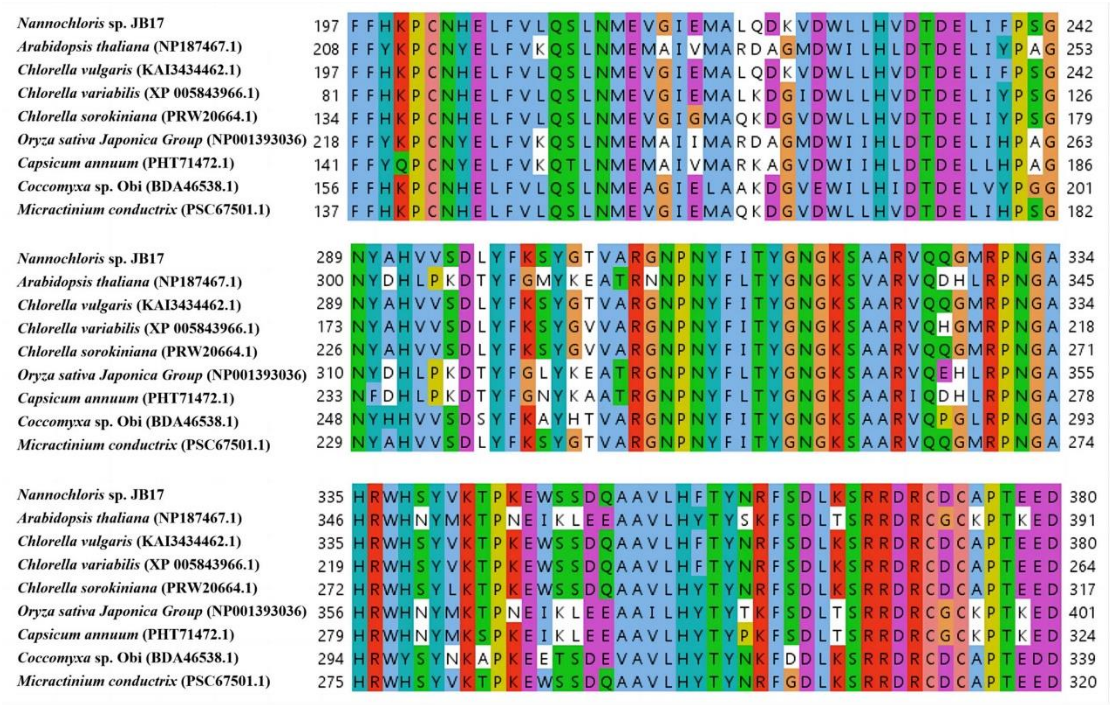

Supplementary Figure 2 Conserved amino acid analysis of glycosyltransferase-like protein KOBITO 1 from different organisms.
